# Prolonged Survival following Repetitive Stereotactic Radiosurgery in a Patient with Intracranial Metastatic Renal Cell Carcinoma

**DOI:** 10.1155/2015/872915

**Published:** 2015-10-27

**Authors:** Ethan A. Ferrel, Andrew T. Roehrig, Wayne T. Lamoreaux, Alexander R. Mackay, Robert K. Fairbanks, Jason A. Call, Jonathan D. Carlson, Benjamin C. Ling, John J. Demakas, Barton S. Cooke, Aaron Wagner, Christopher M. Lee

**Affiliations:** ^1^Gamma Knife of Spokane, 910 W 5th Avenue, Suite 102, Spokane, WA 99204, USA; ^2^Cancer Care Northwest, 910 W 5th Avenue, Suite 102, Spokane, WA 99204, USA; ^3^University of Washington School of Medicine, 1959 NE Pacific Street, Seattle, WA 98195, USA; ^4^Inland Neurosurgery & Spine Associates, 105 W 8th Avenue, Suite 200, Spokane, WA 99204, USA; ^5^Rockwood Clinic, 801 W 5th Avenue, Suite 525, Spokane, WA 99204, USA

## Abstract

Patients with metastatic renal cell carcinoma (RCC) to the brain have a very poor prognosis of three months if left untreated. SRS is an effective treatment modality in numerous patients. This case exemplifies the utility of stereotactic radiosurgery (SRS) in prolonging survival and maintaining quality of life in a patient with RCC. This 64-year-old female patient initially presented to her primary care physician 22 months after a left nephrectomy for RCC with complaints of mild, intermittent headaches and difficulty with balance. An MRI revealed five cerebellar lesions suspicious for intracranial metastasis. The patient's first GKRS treatment targeted four lesions with 22 Gy at the 50% isodose line. She underwent a total of seven GKRS treatments over the next 60 months for recurrent metastases to the brain. 72 months and 12 months have now passed since her brain metastases were first discovered and since her last GKRS treatment, respectively, and this woman is alive with considerable quality of life and no evidence of metastatic reoccurrence. This case shows that repeated GKRS treatments, with minimal surgical intervention, can effectively treat multiple intracranial lesions in select patients, prolonging survival and avoiding iatrogenic neurocognitive decline while maintaining a high quality of life.

## 1. Introduction

Secondary metastasis to the brain continues to be a leading cause of death in cancer patients. Each year in the US, roughly 170,000 new cases of brain metastasis are diagnosed, an estimated 1,200–5,100 of which are secondary to renal cell carcinoma (RCC) [1–5]. Advancements in imaging and treatments of systemic disease have led to an increased prevalence of these tumors across all RCC patients. Due to the very poor prognosis for patients with intracranial RCC, rapid and effective treatment has become vitally important for overall survival and quality of life.

Standard therapies for these tumors include corticosteroids, surgical resection, whole brain radiation therapy (WBRT), and stereotactic radiosurgery (SRS) [6]. Treatment regimens are generally multimodal. Metastases from RCC are notoriously difficult to treat with conventional whole brain radiation due to the radioresistant nature of these tumors [7]. In response, SRS has emerged as an effective alternative, especially for patients with a single metastasis [8]. WBRT is still recommended for patients with >3 metastases, but few studies have examined the effectiveness of treating multiple RCC metastases without adjuvant WBRT [9].

In this report, we discuss the results of a nephrectomized patient with multiple brain metastases from RCC treated with surgery, steroids, and a total of eight GKRS procedures over a 60-month period. At this time, she is alive with no evidence of reoccurrence.

## 2. Case Report

We report on a 64-year-old woman who initially presented to the emergency department with complaints of chest pain following a motor vehicle crash. A CT incidentally found renal abnormalities, and a postnephrectomy biopsy confirmed renal cell carcinoma. The patient was asymptomatic at that time and remained so for ten months, after which she visited her primary care physician with complaints of mild intermittent headaches and difficulty with balance. An MRI revealed five cerebellar lesions measuring 1.4 × 1.5 × 1.6 cm, 2.0 × 1.5 × 1.7 cm, 0.7 × 0.7 × 0.7 cm, 0.2 × 0.2 × 0.2 cm, and 0.1 × 0.1 × 0.1 cm, as well as extensive vasogenic edema.

**Figure 1:**
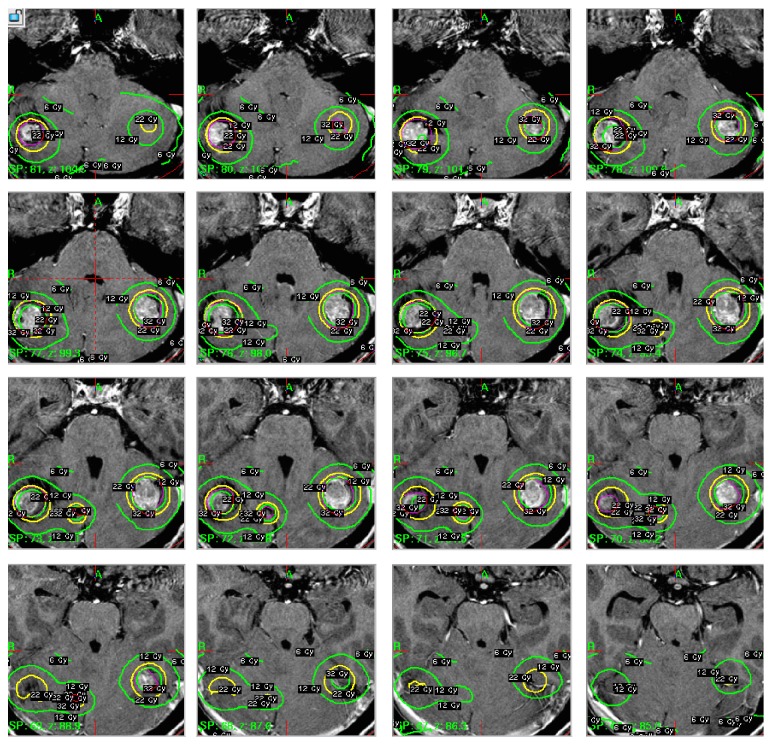
Multiple T-1 postgadolinium axial images illustrating tumor location and Gamma Knife isodose plans (22 Gy, 50% isodose line).

**Figure 2:**
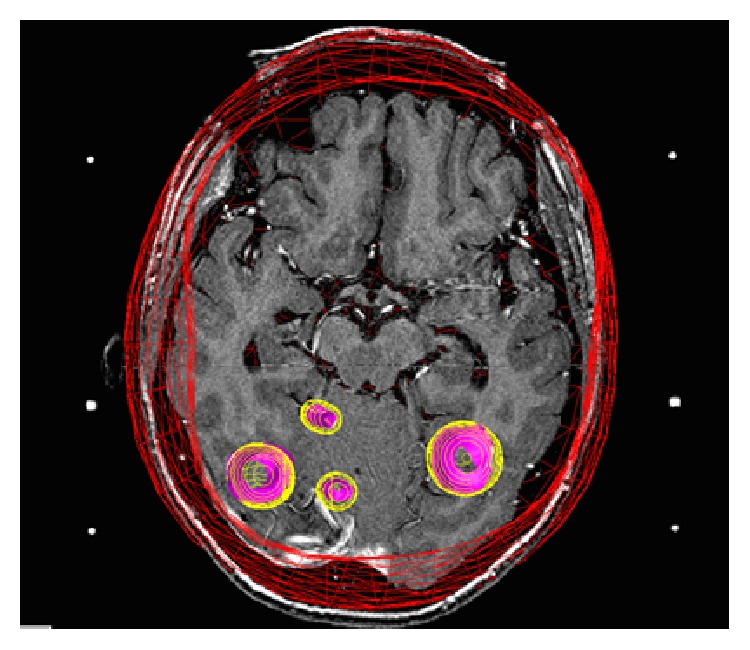
Axial image revealing 3-dimensional wire view of the Gamma Knife radiation dose cloud.

Her treatment team recommended against resection due to the sensitive location of her tumors. GKRS was recommended over WBRT due to concern that avoiding previous radiation fields from treatment for oropharyngeal carcinoma 14 years earlier would not adequately address her relatively caudal metastatic tumors. The patient then underwent her first GKRS treatment at a dose of 22 Gy at the 50% isodose line (Figures 1 and 2). On the day of treatment, her Karnofsky Performance Status score was 100. Following GKRS, she was placed on dexamethasone 2 mg daily. Within several weeks she had experienced significant resolution of symptoms.

Over the next 51 months, she underwent a subsequent six GKRS treatments, all of which treated between one and four metastases. Many of these metastases were in new locations within the cerebellum, while several appeared in the brainstem itself. 49 months after her initial treatment, she underwent a craniotomy to relieve obstructive hydrocephalus with a ventricular shunt placement. An 8.0 mm cerebellar lesion was successfully resected in the same procedure. Around the same time, she underwent SBRT for liver metastases, which was tolerated well.

The patient's most recent GKRS treatment occurred 60 months after the first and was suggested when the patient presented with complaints of consistent falls. A CT demonstrated five lesions, four of which were localized to the cerebellum and one limited to the pons. Three of the lesions had increased in size since her previous CT several months earlier. Three lesions were treated with a dose of 20 Gy at the 50% isodose line, one lesion was treated with 18 Gy at the 75% isodose line, and one was treated with 18 Gy at the 55% isodose line. A posttreatment CT demonstrated reduced dimensions of all lesions. Her most recent CT was taken 5 months later and demonstrated reduced or stable sizes of all lesions. 65 months after her initial diagnosis of brain metastasis, the patient began treatment with Votrient 800 mg for her liver metastases, which was tapered down to 400 mg and eventually held completely for after severe GI side effects and elevated liver enzymes.

One month prior to this paper, a PET scan was obtained, showing no new evidence of systemic or metastatic disease. At that time, she remained symptom-free with a high quality of life a total of 72 months after her brain metastases were diagnosed.

## 3. Discussion

Brain metastasis from renal cell carcinoma remains one of most frequently encountered intracranial secondary cancers, with 4–11% of RCCs spreading to the brain [7]. The poor prognosis of these patients has led to much research into ideal treatment modalities which extend overall survival and maximize quality of life. As new treatments and technologies become more effective in treating the primary disease, intracranial spread is becoming more commonplace [1, 3–5]. Nephrectomy is the first-line treatment for renal cell carcinoma, but a large autopsy study found that nephrectomized patients were not significantly less likely to develop distant metastases than nonnephrectomized patients [10, 11]. Chemotherapeutic treatments, while sometimes effective in treating the primary cancer, seem to have little effect on cerebral tumors [11]. In response, many researchers now focus on how to best address and treat intracranial metastases [9].

### 3.1. Current Treatment Modalities and Benefits of SRS

Much debate exists over the appropriate treatment modalities for these tumors. RCC has long been considered to have radioresistant histology, rendering WBRT of questionable efficacy [11]. A study by Nieder et al. of 336 metastatic lesions treated with WBRT (30 Gy in 10 fractions) showed a complete response rate of 0% for RCC, while small cell carcinoma and breast cancer showed 37% and 35% response rates, respectively [12]. WBRT has also been shown in multiple studies to accelerate neurocognitive decline [13–15]. In a recent study by Brown et al., 91.7% of patients with brain metastasis treated with both SRS and WBRT experienced a cognitive decline compared to 63.5% of patients who underwent SRS alone [15]. Due to these concerns, WBRT is more frequently being recommended only to patients with advanced disease and especially poor prognoses [9].

Surgical resection, while ideal in removing large lesions, is not always capable of treating multiple metastases. Resection could be impractical due to a tumor's location, such as when metastases are distant from each other or lie near critical structures. Metastatic lesions of the brainstem are particularly difficult to resect due to the highly concentrated nuclei and neural tracts. If the tumor can be safely resected, adjuvant radiation to the tumor bed is often applied in order to achieve optimal margins. Important to note is how metastatic RCC is a negative prognostic factor for peritumoral edema [16, 17]. A study by Shuto et al. found that SRS is an effective way of managing symptomatic peritumoral edema especially from small tumors, while removal of a large tumor can be advantageous in relieving severe edema and its symptoms, as was in the case of our patient [16]. Regardless, the invasive and lengthy nature of surgery has rendered faster, less invasive treatments more appealing.

In response to the shortfalls of surgery and WBRT, SRS has proven to be an effective treatment modality [11, 16, 18–20]. SRS is often capable of safely reaching lesions that neurosurgeons cannot and has been shown to minimize the harmful neurocognitive effects of widespread radiation, all while boasting improved local control rates [15, 16, 19]. A retrospective study of metastatic RCC patients treated with SRS alone found that SRS resulted in a local control rate of 94% and median overall survival of 11.4 months [19]. Another study by Sheehan et al. found that patients with metastatic RCC treated with SRS alone experienced a local control rate of 96% and median overall survival of 15 months [18]. In addition to being effective, SRS is a rapid and minimally invasive procedure with a low rate of complications that can often be completed in a single treatment. A study by Hong et al. analyzed adverse effects of 279 SRS procedures and found that <2% of patients experienced sequelae requiring hospitalization, and 34% experienced mild to moderate side effects [21]. SRS has repeatedly demonstrated its utility in treating brain metastases and ought to be considered a first-line therapy for patients with positive prognostic factors.

### 3.2. Prognostic Factors and SRS Dosing Considerations

Many studies have attempted to determine prognostic factors for patients with brain metastasis. Currently, the Radiation Therapy Oncology Group's (RTOG) recursive partitioning analysis (RPA) system is used most frequently to establish prognosis [22]. The RPA categorizes patients into three classes based on their Karnofsky Performance Score (KPS), age, number of extracranial metastases, and status of primary cancer, where a higher RPA class indicates a worse prognosis [1, 3–5]. Muacevic et al. determined that a KPS > 70 and RPA Class 1 were correlated to a better survival prognosis [19]. A sizeable study by Kano et al. found that number of intracranial metastases is a significant prognostic factor as well as age and KPS, while a study by Powell et al. found that KPS was a better prognostic indicator than number of intracranial metastases [20, 23]. These observations are essential in establishing treatment guidelines for physicians. Tailored treatment plans of modality, dosages, and other factors require such insights to achieve optimal outcomes in individual patients.

SRS dosage also seems to be an important factor in the treatment of intracranial RCC. A 2015 study by Rades et al. determined that treatment with 20 Gy results in better 12-month survival rates than 16–18 Gy at 81% and 50%, respectively, in patients with brain metastatic RCC [24]. To date, this is the only study on the impact of dose on survival in patients with RCC treated solely with SRS. Lorenzoni et al. found that a dose higher than 18 Gy was associated with longer survival in a multivariate analysis of patients with intracranial tumors from a variety of cancers treated with SRS [25]. Further research on the effect of SRS dose on overall survival is necessary to understand this potentially important correlation.

## 4. Conclusion

Our report demonstrates the efficacy of treating multiple metastases from renal cell carcinoma with SRS without adjuvant WBRT. Few reported cases have demonstrated such extended survival times of patients with metastatic RCC treated with SRS alone. This patient demonstrates extended survival and high quality of life after undergoing repeated GKRS treatments. 72 months after the diagnosis of her brain metastases, she is alive with an appreciable quality of life. SRS ought to be strongly considered as the primary treatment for multiple intracranial metastases in RCC patients.
